# Identification of Critical Molecular Pathways Induced by HDAC11 Overexpression in Cardiac Mesenchymal Stem Cells

**DOI:** 10.3390/biom15050662

**Published:** 2025-05-03

**Authors:** Chongyu Zhang, Neal L. Weintraub, Yaoliang Tang

**Affiliations:** Vascular Biology Center, Department of Medicine, Medical College of Georgia, Augusta University, 1460 Laney Walker Blvd, Augusta, GA 30912, USA; chozhang@augusta.edu (C.Z.); nweintraub@augusta.edu (N.L.W.)

**Keywords:** HDAC11, cardiac mesenchymal stem cell, RNA-seq, lncRNA

## Abstract

HDAC11, the only class IV histone deacetylase, primarily functions as a fatty acid deacylase and has been implicated in metabolic regulation, cancer stemness, and muscle regeneration. However, its role in cardiac mesenchymal stem cells (CMSCs) remains unexplored. To investigate the effects of HDAC11 overexpression on the gene regulatory networks in CMSCs, we treated mouse CMSCs with an adenoviral vector encoding human HDAC11 (Ad-HDAC11) versus adenoviral GFP (Ad-GFP) as a control. Gene expression and pathway enrichment were assessed using RNA sequencing (RNA-seq), and HDAC11 overexpression was validated at the RNA and protein levels through qRT-PCR and Western blot. RNA-seq and Gene Ontology (GO) analysis revealed that HDAC11 overexpression activated cell cycle pathways while suppressing nucleotide transport and phagolysosome-related processes. Furthermore, pHH3 protein level was increased, suggested enhanced proliferation in HDAC11-overexpressed CMSCs. qRT-PCR also confirmed the downregulation of GM11266, a long non-coding RNA, in HDAC11-overexpressing CMSCs. In summary, HDAC11 overexpression promotes transcriptional reprogramming, cell cycle progression, and CMSC proliferation, underscoring its potential role in regulating CMSC growth and division.

## 1. Introduction

Histone deacetylases (HDACs) broadly function in removing acetyl groups from ε-N-acetyl lysine on histone and non-histone proteins, thereby regulating protein function and condensing the chromatin structure to inhibit gene transcription [1,2]. There are two main families of HDACs: the Zn2+-dependent HDACs and NAD+-dependent HDACs. The Zn2+-dependent HDACs can be further categorized into three classes based on their active domain homology: classes I, II, and IV [1]. HDAC11 was first discovered in 2002 and is the only class IV HDAC [3]. What distinguishes HDAC11 from the other Zn2+-dependent HDACs is that it has virtually no detectable deacetylase activity, while its fatty acid deacylase activity is reportedly 10,000 times stronger than its deacetylase activity [4], although findings have been inconsistent [5]. HDAC11 can be activated by normal levels of free fatty acids and plays an essential role in metabolism [6]. In obesity, HDAC11 regulates the adipocyte phenotype through the demyristoylation of gravin-α and suppression of β-adrenergic receptor signaling [7]. It also indirectly suppresses thermogenic genes in adipose tissue by binding to a transcriptional repressor, BRD2, and preventing active histone modifications [8]. Moreover, HDAC11 has been reported to function in numerous physiological and pathological processes, including immune response, cell cycle, metabolism, ischemic injury, and tumor biology [9,10,11,12,13,14,15].

The functions of HDAC11 vary widely depending on the context and cell types [14]. In breast cancer, HDAC11 has been reported to inhibit cancer proliferation and metastasis [16]. However, in hepatocellular carcinoma, HDAC11 expression was elevated and associated with poor patient outcomes, while its overexpression promoted cancer stemness and resistance to sorafenib therapy [5]. Conversely, in fibroblasts, HDAC11 overexpression inhibited cell cycle progression in both transformed and non-transformed fibroblasts [17], while in age-related macular degeneration, HDAC11 overexpression reduced chromatin accessibility [18]. HDAC11 inhibits replication of the hepatitis B virus by the suppression of cccDNA transcription through inhibiting the acetylation of cccDNA [19]. In stem cells, HDAC11 reportedly has divergent effects depending on the tissue type. In bovine and murine skeletal muscle stem cells, HDAC11 activates the Notch signaling pathway to promote muscle stem cell proliferation while inhibiting stem cell differentiation, and ultimately muscle regeneration [20,21]. Similarly, in white adipose tissue, Yang et al. [22] found that HDAC11 levels were elevated in obese individuals, and its repression promoted a shift in the fate of adipose-derived stem cells toward the brown adipocyte phenotype [8,13].

HDAC11 is involved in transcriptional regulation and cell cycle progression [17,19,23] and appears to play an important role in stem cell fate. In our previous work, we identified mouse cardiac mesenchymal stem cells (CMSCs) as GATA4-positive mesenchymal stem cells [24]. Mesenchymal stem cells (MSCs), including CMSCs, are known to secrete pro-angiogenic cytokines such as VEGF, bFGF, and SDF-1α, which contribute to angiogenesis and paracrine-mediated cardiac repair in ischemic myocardium [25,26]. We have also demonstrated that exosomes derived from cardiac MSCs enhance angiogenesis and improve cardiac function following acute myocardial infarction [27]. After myocardial injury, endogenous cardiac stem cells, including CMSCs, are rapidly depleted in both infarcted and non-infarcted regions [28], limiting their reparative capacity. HDAC11 has been shown to promote muscle stem cell proliferation through activation of the Notch signaling pathway, while concurrently suppressing differentiation, thus expanding the stem cell pool. Although its function in cardiac stem cells remains unknown, based on these findings, we hypothesized that HDAC11 overexpression in CMSCs may enhance their proliferative capacity, potentially amplifying their regenerative potential and improving cardiac repair outcomes. In this study, we overexpressed HDAC11 in CMSCs to explore its effects on the gene regulatory networks.

## 2. Materials and Methods

### 2.1. Isolation of Mouse CMSCs

Hearts were harvested from two-month-old male C57/BL6 mice (The Jackson Laboratory, Bar Harbor, ME, USA). After rinsing with 1× phosphate-buffered saline (PBS), CMSCs were isolated following established protocols [29,30]. In brief, hearts were minced into approximately one mm^3^ pieces and enzymatically digested with 0.1% collagenase IV and 1 U/mL dispase in Dulbecco’s Modified Eagle Medium F-12 (DMEM/F-12) at 37 °C for 30 min. The digested cardiac explants were then seeded onto fibronectin/gelatin-coated plates (0.5 mg fibronectin in 100 mL of 0.1% gelatin solution) in complete DMEM media containing 10% fetal bovine serum (FBS), 100 U/mL penicillin G, and 100 μg/mL streptomycin. The cells were cultured under these conditions until confluent within 7–10 days. Lin^−^Sca-1^+^ cells were subsequently isolated from the cardiac-derived cells using a hematopoietic Lin-depletion cocktail (StemCell Technologies, Vancouver, BC, Canada) followed by magnetic sorting with an anti-Sca-1-microbead kit (Miltenyi Biotec), following the manufacturer’s instructions. The Lin^−^Sca-1^+^ cells, referred to as CMSCs, were maintained in complete DMEM media. CMSCs were passaged up to 10 times at 5-day intervals to ensure consistent cell quality.

### 2.2. Adenovirus Transduction

CMSCs were cultured to 70% confluency and then treated with either Ad-GFP [31] or Ad-HDAC11, which contains the open reading frame (ORF) of human HDAC11 with a C-terminal Flag and His tag (ViGene Biosciences, Rockville, MD, USA), at a multiplicity of infection (MOI) of 500 for 48 h.

### 2.3. RNA Extraction, Next-Generation Sequencing, and Bioinformatic Analysis

Following the manufacturer’s protocol, total RNA was extracted from CMSC cells using RNAZol RT (Molecular Research Center, OH, USA). The RNA was resuspended in RNase/DNase-free water for downstream analysis. RNA sequencing (RNA-seq) analysis was performed by Novogene (Sacramento, CA, USA). Read counts for each treatment group (*n* = 2 replicates per group) were generated using Arraystar software v.17.2.1 (DNASTAR, Madison, WI, USA), and differentially expressed genes (DEGs) were identified with DESeq2 analysis, comparing Ad-HDAC11-treated CMSCs to Ad-GFP-treated CMSCs. DEGs with an average log2 fold change (avg_log2FC) > 1 and adjusted *p*-value (*p*_val_adj) < 0.05 were considered significantly different. Volcano plots were created using the “EnhancedVolcano” package (v1.14) in R Studio (version 1.1.456). Gene Ontology (GO) pathway enrichment analysis and Gene Set Enrichment Analysis (GSEA) were performed on the DEGs using the gseGO function from the R/Bioconductor package “clusterProfiler” (v4.4.4) with org.Mmu.eg.db (v3.15). GO terms with *p* < 0.05 were considered as significant. GO enrichment results were visualized as bubble plots, and the most enriched gene networks were illustrated using the cnetplot function.

### 2.4. Real Time qRT-PCR

The RevertAid RT kit (ThermoFisher, Waltham, MA, USA) was used to reverse transcribe RNA into cDNA. Real-time qPCR was conducted on the CFX96 system (BioRad, Hercules, CA, USA) using PowerUp SYBR Green Master Mix following the manufacturer’s instructions (ThermoFisher). Data analysis was performed using the ΔΔCT method.

Primers used:
hHDAC11:F primer: AGGGCTACCATCATTGATCTTGR primer: GTAGATGTGGCGGTTGTAGACSELP:F primer: AGTTTCCGGTTCCCAGTAAAGR primer: CCAGTAGCCAGGCATCTTATCFABP3:F primer: GACCAAGCCTACTACCATCATCR primer: GTCACCTCGTCGAACTCTATTCGM11266:F primer: GTAATGAAGCCAACCAGAAGAAAGR primer: CCAAGTCCAGGTAACCAGTATGGM16548:F primer: GAGCATTGTGGTTTGCTTTGAR primer: CTGTGTGTGACTCTTGGGTATGGAPDH:F primer: CAAATGGCAGCCCTGGTR primer: CCTCGTCCCGTAGACAAA

### 2.5. Western Blot

Western blotting was performed as previously described [32]. Briefly, protein lysates were separated on a 10% SDS-PAGE gel and transferred onto a nitrocellulose membrane (LI-COR) for immunoblotting. The membrane was incubated overnight at 4 °C with primary antibodies: rabbit anti-phospho-histone H3 Ser10 (pHH3) (1:1000, #06-570, Upstate Cell Signaling Solutions, Lake Placid, NY, USA), rabbit anti-HDAC11 (1:1000, #NBP2-16789, Novus Biologicals, Minneapolis, MN, USA), and mouse anti-β-actin (1:5000, #NB600-501, Novus Biologicals, Minneapolis, MN, USA). After washing with 1× TBST, the membrane was incubated for 1 h at room temperature with secondary antibodies, including IRDye 680 goat anti-rabbit IgG and IRDye 800 goat anti-mouse IgG (1:10,000, LI-COR Biosciences, Lincoln, NE, USA). Detection was performed using an Odyssey infrared imaging system (LI-COR Biosciences).

### 2.6. Measuring Nuclear Size Using Fluorescent Staining

To compare nuclear size relative to total cell size, CMSCs were stained with a PKH26 Red Fluorescent Cell Linker Kit (#PKH26GL, Sigma, St. Louis, MO, USA) and incubated overnight; the nuclei were subsequently stained with Hoechst33342 (#R37605, ThermoFisher) for 15 min. Following staining, cells were imaged using an EVOS FL microscope (ThermoFisher) to assess nuclear and cell size.

### 2.7. Statistical Analysis

All data are presented as the mean ± SEM. The two groups were compared using an unpaired Student’s *t*-test (GraphPad Prism, version 9.41), with *p*-values ≤ 0.05 considered statistically significant.

## 3. Results

### 3.1. Confirmation of HDAC11 Overexpression in CMSCs

**Figure 1:**
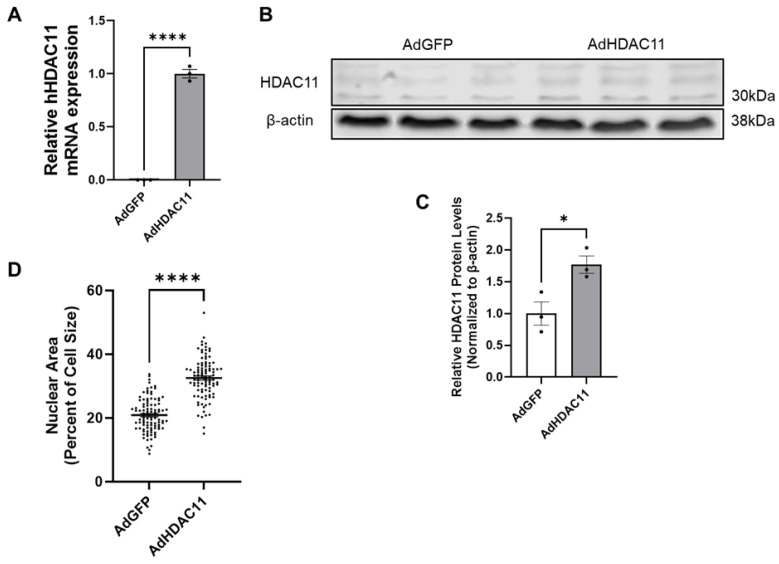
HDAC11 is successfully overexpressed in CMSCs following Ad-HDAC11 transduction. (**A**) Relative mRNA expression of hHDAC11 is significantly higher in the Ad-HDAC11 group compared to Ad-GFP controls, as assessed by qRT-PCR. There is almost no detectable expression of human HDAC11 in the Ad-GFP group. (**B**,**C**) Western blot analysis shows that HDAC11 protein levels, normalized to β-actin, are significantly increased in Ad-HDAC11-transduced CMSCs compared to Ad-GFP controls, confirming the successful overexpression of the human HDAC11 gene in mouse CMSCs (*n* = 3) (Original images can be found in the Supplementary Materials). (**D**) Nuclear size and total cell size were quantified using ImageJ 1.54f. Nuclear area as a percentage of total cell size is presented (*n* = 100). Data are presented as the mean ± SEM. * *p* ≤ 0.05, **** *p* ≤ 0.0001 by Student’s *t*-test.

We transduced mouse CMSCs with adeno-human HDAC11 (Ad-HDAC11) or Ad-GFP as a control. RNA and protein were collected for analysis. qRT-PCR was performed using primers specific for human HDAC11, which resulted in almost no detectable expression in Ad-GFP-treated CMSCs. In contrast, human HDAC11 RNA levels were significantly increased in Ad-HDAC11-treated cells (Figure 1A). Western blot analysis further confirmed a significant increase in HDAC11 protein levels in Ad-HDAC11-treated CMSCs compared to controls (Figure 1B,C). Interestingly, we also found that the CMSCs treated with Ad-HDAC11 had significantly larger nuclei compared to the control group (Ad-GFP-treated CMSCs) (Figure 1D). An increased nucleus-to-cytoplasm (N/C) ratio indicates enhanced nuclear content (e.g., DNA) relative to cytoplasmic volume. This shift is often linked to mechanisms such as mitotic chromatin regulation and DNA replication during the S phase of the cell cycle [33,34].

### 3.2. Differential Gene Expression and Functional Enrichment Analysis of HDAC11-Overexpressing Versus Control CMSCs

**Figure 2:**
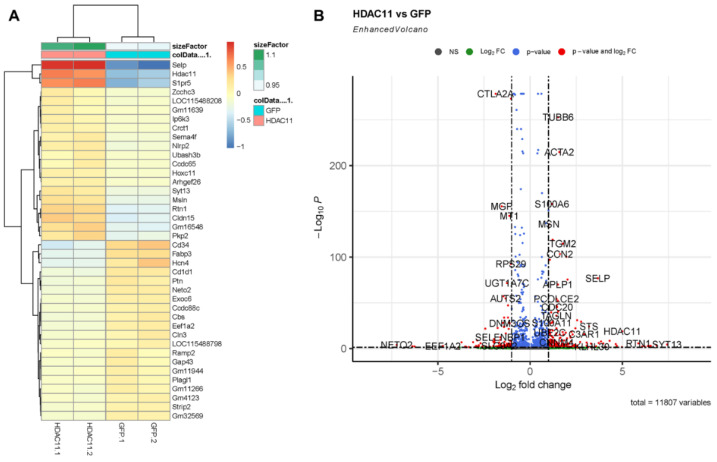
Differential gene expression in Ad-GFP- and Ad-HDAC11-transduced CMSCs. (**A**) A heatmap displays the most significant differentially expressed genes (DEGs) between Ad-GFP- and Ad-HDAC11-transduced CMSCs. The heatmap shows distinct clustering of genes based on expression patterns, with a clear separation between Ad-GFP and Ad-HDAC11 groups. (**B**) A volcano plot highlights the DEGs between the two groups. Genes with significant differential expression are represented as red dots. Upregulated genes in the Ad-HDAC11 group are shown on the right side of the plot, while downregulated genes are displayed on the left side. Notably, HDAC11 was one of the most highly upregulated genes detected in Ad-HDAC11-transduced CMSCs, confirming HDAC11 overexpression.

We performed RNA-seq on CMSCs transduced with either the GFP control or HDAC11-overexpressing adenovirus. The heatmap of differentially expressed genes (DEGs) confirmed the successful overexpression of HDAC11 in the Ad-HDAC11 group (Figure 2A).

The volcano plot analysis highlights significant changes in the gene expression of HDAC11-overexpressing CMSCs (Figure 2B). Genes such as TUBB6, ACTA2, SELP, and HDAC11 were markedly upregulated in HDAC11-overexpressing CMSCs. In contrast, genes including CTLA2A were significantly downregulated, suggesting that HDAC11 overexpression induced extensive transcriptional changes including genes involved in diverse cellular functions.

### 3.3. Functional Enrichment Analysis of GO Biological Processes in HDAC11-Overexpressing CMSCs

**Figure 3:**
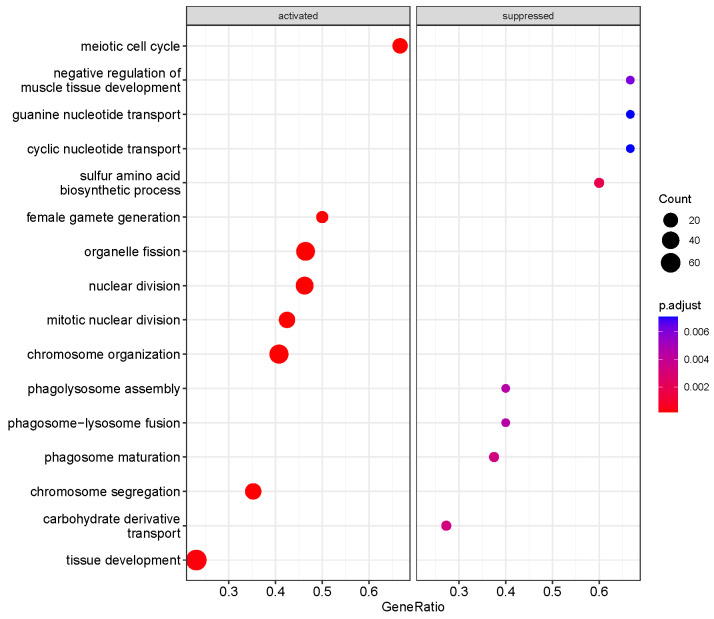
Functional enrichment analysis of differentially expressed genes between Ad-HDAC11- and Ad-GFP-transduced CMSCs identified key Gene Ontology biological process (GO-BP) terms. The dot size represents the number of genes associated with each pathway, and the color gradient indicates the significance of enrichment, with red denoting the most significant pathways.

We performed a GO biological process (BP) enrichment analysis using the clusterProfiler package to identify pathways altered in HDAC11-overexpressing CMSCs (Figure 3). Several significantly activated pathways were associated with cell cycle regulation, including processes such as meiotic cell cycle, mitotic nuclear division, nuclear division, chromosome organization, and chromosome segregation. Additionally, pathways related to tissue development were also upregulated. In contrast, the most significantly suppressed pathways in HDAC11-overexpressing CMSCs were primarily related to nucleotide transport (e.g., cyclic and guanine nucleotide transport) and phagolysosome function (e.g., phagolysosome assembly, phagosome–lysosome fusion, and phagosome maturation). These findings suggest that HDAC11 overexpression drives cellular processes involved in growth and selective tissue development while suppressing intracellular transport dynamics.

### 3.4. Chromosome Organization Is a Major Activated Pathway in HDAC11-Overexpressing CMSCs

**Figure 4:**
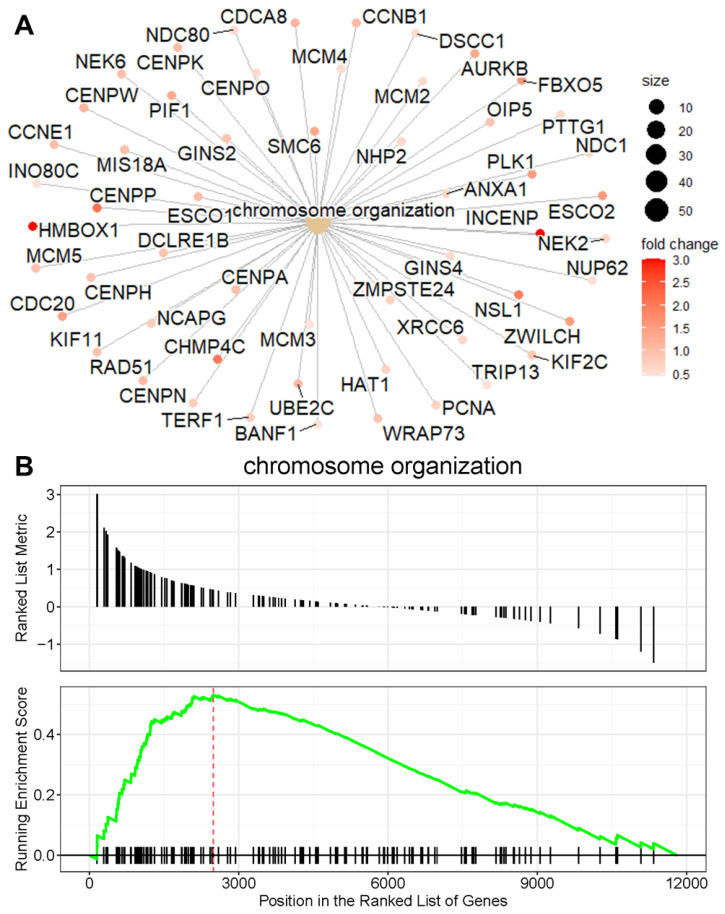
Chromosome organization is a major activated pathway in HDAC11-overexpressing CMSCs. (**A**) The cnetplot shows the network of genes associated with the chromosome organization pathway, highlighting their interconnections. The node size corresponds to gene connectivity within the pathway, and the color gradient reflects the fold change in expression, with red representing the highest fold change. (**B**) The GSEA plot demonstrates the enrichment of the chromosome organization gene set. The running enrichment score (green line) peaks at the leading-edge subset (indicated by the red dashed line), showing a significant overrepresentation of chromosome organization genes at the top of the ranked gene list. Black tick marks below the plot indicate the positions of individual genes within the pathway.

To better understand the relationship between enriched pathways and the involved genes, we employed a cnetplot for visualization. This approach highlights the genes contributing to the enriched pathways and their annotation categories. Figure 4A illustrates the network of the activated chromosome organization pathway, which includes upregulated genes such as AURKB, PCNA, INO80C, and HMBOX1 in HDAC11-overexpressing CMSCs. In brief, AURKB (Aurora B) plays a central role in mitosis and chromosomal segregation. Its deletion disrupts cell division and can impair embryonic development [35]. PCNA (proliferating cell nuclear antigen) functions in DNA replication and repair by forming a sliding clamp that tethers polymerases to DNA and recruits other repair proteins through its PCNA interacting protein (PIP)-box motif [36]. INO80C (INO80 complex subunit C) is part of a chromatin remodeling complex that stabilizes replication forks and downregulates the S-phase checkpoint, ensuring smooth DNA replication [37]. HMBOX1 (homeobox containing 1) is a multifunctional factor that supports the endothelial differentiation of bone marrow stromal cells, suppresses inflammation in hepatocytes and NK cells, and exhibits context-dependent roles in cancer progression [38]. These findings were further validated by gene set enrichment analysis (GSEA) (Figure 4B), which identified chromosome organization as the major enriched gene set in HDAC11-overexpressing CMSCs compared to GFP controls. The running enrichment score curve shows a strong accumulation of chromosome-organization-related genes at the top of the ranked list, confirming their significant upregulation. Together, these results demonstrate that HDAC11 overexpression drives the activation of genes involved in chromosome organization, further supporting its role in regulating processes essential for cell division and nuclear dynamics.

### 3.5. Identification of Hub Genes in HDAC11-Overexpressing CMSCs

**Figure 5:**
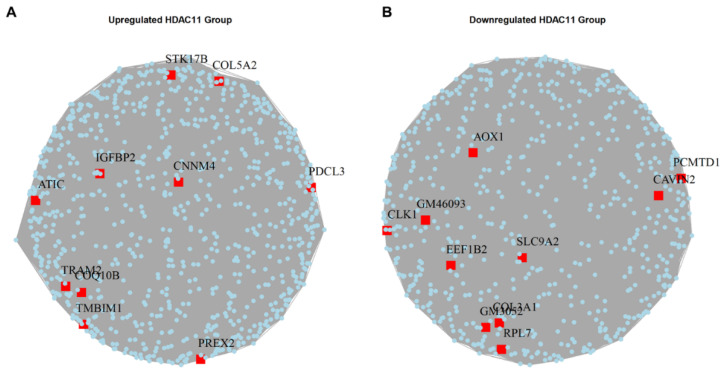
Hub genes in the upregulated and downregulated gene sets of Ad-HDAC11-transduced CMSCs compared to Ad-GFP controls. (**A**) The upregulated hub genes in HDAC11-overexpressing cells. (**B**) The downregulated hub genes in HDAC11-overexpressing cells.

Hub genes, which interact with multiple other genes within a gene network, play central roles in biological processes by influencing numerous downstream pathways. To identify these hub genes, we utilized the igraph package to generate a graph object and analyze differentially expressed genes in HDAC11-overexpressing versus control CMSCs [39]. Figure 5A highlights the top upregulated hub genes, including (1) STK17B (DRAK2), a downstream effector of mGluR1–PKCγ signaling, linked to apoptosis and immune function [40]; (2) COL5A2—encodes the α2-chain of type V collagen, a key structural component of the extracellular matrix [41]; (3) IGFBP2 (insulin-like growth factor binding protein 2)—a developmental regulator that modulates IGF activity in the pericellular space, highly expressed in embryonic tissues [42]; (4) CNNM4—a basolateral magnesium transporter; knockout leads to hypomagnesemia due to intestinal Mg^2+^ malabsorption [43]; (5) PDCL3 (phosducin-like 3)—stabilizes VEGFR-2 by binding its juxtamembrane domain and inhibiting its ubiquitination and degradation [44]; (6) ATIC (5-aminoimidazole-4-carboxamide ribonucleotide formyltransferase/IMP cyclohydrolase)—a bifunctional enzyme catalyzing the final steps of de novo purine biosynthesis [45]; (7) TRAM2—a mediator of YAP-driven oncogenic proliferation, correlating strongly with YAP activity [46]; (8) COQ10 (Coenzyme Q10)—involved in mitochondrial electron transport and ATP production, enhances antioxidant activity [47]; (9) TMBIM1 (Transmembrane BAX inhibitor motif-containing 1)—regulates the multivesicular body (MVB) –lysosomal pathway and promotes TLR4 degradation via the ESCRT complex [48]; and (10) PREX2 (phosphatidylinositol-3,4,5-triphosphate-dependent Rac exchange factor 2)—an oncogene that inhibits the activity of phosphatase and tensin homolog (PTEN), contributing to tumorigenesis [49].

Figure 5B shows the downregulated hub genes, which include (1) AOX1 (aldehyde oxidase 1)—produces hydrogen peroxide; its overexpression reduces NADP, PPP flux, and tumor cell invasion [50]; (2) PCMTD1 (protein-l-isoaspartate O-methyltransferase domain-containing protein 1)—a putative E3 ligase adaptor that associates with Cullin-RING complexes [51]; (3) CAVIN2 (caveolae associated protein 2)—a tumor suppressor that induces G2/M arrest and inhibits cell proliferation [52]; (4) CLK1 (Cdc2-like kinase 1)—modulates alternative splicing by phosphorylating serine-arginine rich (SR) proteins essential for spliceosome function [53]; (5) EEF1B2 (eukaryotic translation elongation factor 1 beta 2)—a translation elongation factor that aids tRNA delivery to ribosomes via eEF1A interaction [54]; (6) SLC9A2 (solute carrier family 9 member A2)—a sodium-hydrogen exchanger isoform that suppresses aerobic glycolysis and tumor invasion [55]; (7) COL3A1 (collagen type III alpha 1 chain)—an extracellular matrix protein prevalent in skin, muscle, and blood tissues [56]; and (8) RPL7 (ribosomal protein L7)—a critical 60S ribosomal assembly factor involved in translation regulation [57].

These hub genes might potentially mediate key cellular changes in the transcriptional reprogramming induced by HDAC11 overexpression.

### 3.6. The Effects of HDAC11 Overexpression on CMSC Proliferation

**Figure 6:**
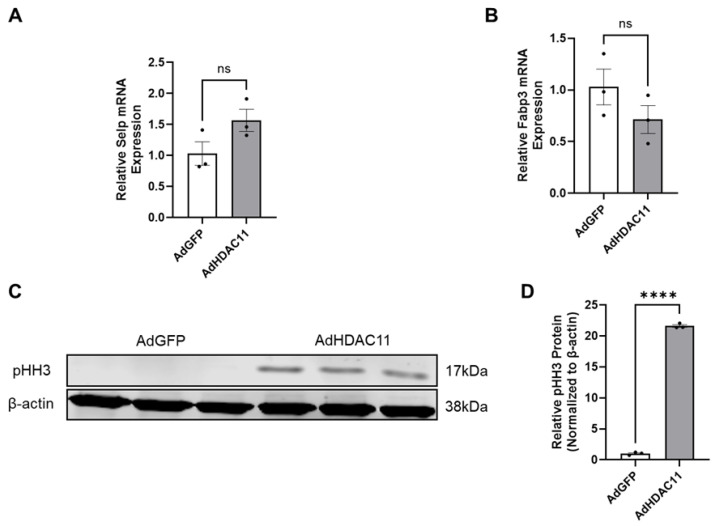
Validation of key HDAC11-regulated genes and mitotic activity. (**A**,**B**) qRT-PCR quantification of the expression of selected genes in HDAC11-overexpressing CMSCs compared to Ad-GFP controls; data bars represent the mean ± SEM (*n* = 3), ns: no significant difference. (**C**,**D**) Western blot quantification of phosphorylated histone H3 (pHH3), a marker of mitotic activity, in HDAC11-overexpressing CMSCs compared to Ad-GFP controls (Original images can be found in the Supplementary Materials). **** *p* ≤ 0.0001 by a Student’s *t*-test.

We validated several of the most differentially expressed genes identified by RNA-seq using qRT-PCR. Specifically, we selected SELP for validation as an upregulated mRNA because it was among the top-two most upregulated genes (excluding HDAC11) in the heatmap shown in Figure 2A. Similarly, we chose FABP3 as a representative downregulated gene, as it was one of the top-two most downregulated mRNAs in the same heatmap. In addition to its strong differential expression, FABP3 is biologically relevant due to its role in the uptake, intracellular transport, and metabolism of long-chain fatty acids [58]. Given that HDAC11 activity is influenced by normal levels of free fatty acids and is implicated in metabolic regulation, the selection of FABP3 also aligns with our interest in exploring the metabolic consequences of HDAC11 overexpression. The results indicated that SELP mRNA levels showed an increasing trend (Figure 6A), while FABP3 mRNA levels exhibited a decreasing trend (Figure 6B). To evaluate the effect of HDAC11 overexpression on cell proliferation, we analyzed pHH3, a marker of M-phase. As shown in Figure 6C,D, HDAC11-overexpressing CMSCs exhibited significantly increased protein level of pHH3 compared to Ad-GFP controls, indicating enhanced cell proliferation upon HDAC11 overexpression. These results suggest HDAC11 overexpression promotes cell proliferation in CMSCs.

### 3.7. HDAC11-Targeted Long Non-Coding RNAs (lncRNAs)

**Figure 7:**
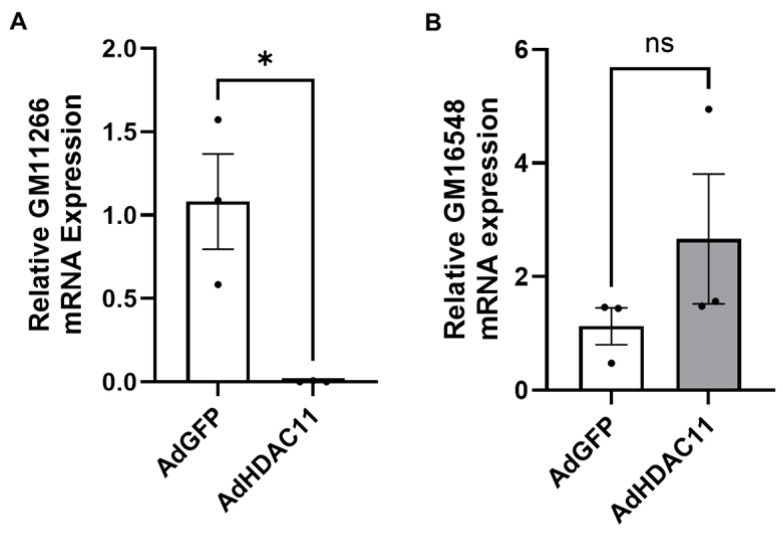
Validation of differentially expressed lncRNAs by qRT-PCR in CMSCs. (**A**) GM11266 expression was significantly downregulated in Ad-HDAC11-transduced CMSCs compared to Ad-GFP controls (* *p* ≤ 0.05). (**B**) GM16548 expression showed an increasing trend in Ad-HDAC11-transduced cells but was not statistically significant (ns). Data are presented as the mean ± SEM (*n* = 3). Statistical analysis was performed using a Student’s *t*-test.

Our RNA-seq analysis identified the dysregulation of lncRNAs in response to HDAC11 overexpression; GM11639 and GM16548 were upregulated, while GM11944, GM11266, GM4123, and GM32569 showed downregulated expression. The downregulation of GM11266 in Ad-HDAC11-treated CMSCs was validated by qRT-PCR, while there was a strong trend toward the upregulation of GM16548 (Figure 7).

## 4. Discussion

Our study demonstrates that HDAC11 overexpression in cardiac mesenchymal stem cells induces significant transcriptional reprogramming, promoting cell cycle progression and enhancing cell proliferation. RNA-seq analysis revealed the upregulation of genes involved in chromosome organization and cell cycle pathways, including meiotic cell cycle, mitotic nuclear division, nuclear division, chromosome organization, and chromosome segregation. In contrast, pathways associated with nucleotide transport (e.g., cyclic and guanine nucleotide transport) and phagolysosome function (e.g., phagolysosome assembly, phagosome–lysosome fusion, and phagosome maturation) were notably downregulated. Using cnetplot analysis, we observed the activated chromosome organization network, highlighting upregulated genes such as AURKB, PCNA, INO80C, and HMBOX1 in HDAC11-overexpressing CMSCs. A hub gene analysis further showed the key upregulated hub genes, including PREX2, CNNM4, PDCL3, STK17B, ATIC, TRAM2, and IGFBP2, many of which are involved in cell proliferation (e.g., TRAM2, PREX2). Conversely, we identified downregulated hub genes such as PCMTD1, RPL7, SLC9A2, CAVIN2, AOX1, CLK1, EEF1B2, and GM46093, many of which are involved in oxidative stress response (e.g., AOX1) and protein synthesis (e.g., RPL7, EEF1B2). Additionally, HDAC11 overexpression altered lncRNA expression, with GM11639 and GM16548 upregulated, while GM11944, GM11266, GM4123, and GM32569 were downregulated. To validate the impact on proliferation, a Western blot assay showed increased levels of phosphorylated histone H3 (pHH3), indicating increased mitotic activity in HDAC11-overexpressing CMSCs. Together, these findings highlight HDAC11’s role in driving cell cycle regulation, chromosomal organization, and CMSC proliferation, suggesting that its overexpression may affect the regenerative capacity of CMSCs.

HDAC11 is a class IV HDAC that has stronger fatty acid deacylase activity than deacetylase activity [4]. Its functions have not been as well-studied as other HDAC family members. HDAC11 has mainly been implicated in metabolic diseases and various cancers [12,13,14]. Higher levels of HDAC11 are associated with obesity, cancer stemness, and muscle regeneration [13,21,59].

RNA-seq analysis shows that the overexpression of HDAC11 highly increased the RNA expression of SELP and S1PR5, both of which are involved in cell proliferation and differentiation [60,61]. Additional upregulated genes might support HDAC11’s role in cell proliferation and differentiation. For instance, NLRP2 inhibits NF-κB activity and regulates iPSC differentiation [62], while PKP2 regulates cardiac electrical functions and cardiac development [63]. Notably, since HDACs typically function as gene suppressors, the downregulated genes in HDAC11-overexpressing CMSCs are particularly noteworthy. Major downregulated genes include CD34, essential for stem cell attachment [64,65]; FABP3, which mediates the intracellular transport of long-chain fatty acids and inhibits stem cell proliferation [66]; HCN4, which encodes a potassium channel vital for native pacemaker currents in the heart [67]; PTN, known to regulate both cell proliferation and differentiation [68,69]; and CCDC88C, required for G-protein activation in Wnt signaling and driving cancer metastasis [70]. Moreover, HDAC11 overexpression markedly increased the expression of tubulin beta 6 class V (TUBB6) and smooth muscle actin alpha 2 (ACTA2), major components of microtubules and actin essential for cell structure and mobility. Concurrently, HDAC11 overexpression decreased levels of matrix Gla Protein (MGP) and metalloproteinase membrane type 1 (MT1). Additionally, genes such as EEF1A2 (protein synthesis and cancer cell survival) [71], RAMP2 (cardiovascular homeostasis) [72], PLAGL1 (cell growth suppression and differentiation activation) [73], and STRIP2 (stem cell differentiation and cell morphology regulation) [74] were also suppressed. Together, these findings suggest that HDAC11 overexpression represses genes critical for stem cell and cardiac functions while promoting those associated with proliferation and differentiation in CMSCs. Increased pHH3 protein levels further confirm CMSCs undergoing mitosis in response to HDAC11 overexpression.

Unlike skeletal muscle stem cells [21], HDAC11 overexpression in CMSCs prominently upregulated genes associated with chromosome organization. This includes the significant upregulation of homeobox containing 1 (HMBOX1) and NIMA related kinase 2 (NEK2) (Figure 4A). HMBOX1 plays a critical role in regulating telomerase activity for telomere elongation and participates in DNA damage responses, while NEK2 is essential for centrosome separation, bipolar spindle formation in mitotic cells, and chromatin condensation in meiotic cells [75,76]. Additionally, other cell-cycle-related pathways were upregulated by HDAC11 overexpression, as evidenced by the upregulation of genes such as AURKB and PCNA, which regulate critical steps of cell division. These findings underscore the role of HDAC11 in driving pathways essential for chromosome organization and cell cycle progression in CMSCs.

Gene Ontology pathway analysis revealed that HDAC11 overexpression suppressed pathways related to phagosome–lysosome formation and nucleotide transport. This suppression is highlighted by the downregulation of key lysosomal function genes, such as TMEM175 and CLN3, as well as critical transporter genes, including SLC19A1 and ABCC5. Additionally, our analysis showed that some upregulated hub genes in HDAC11-overexpressing cells, such as PREX2, CNNM4, STK17B, and IGFBP2, are associated with cell cycle regulation and proliferation. This aligns with previous reports that implicate HDAC11 in driving cell proliferation, especially in various types of cancers [14].

Many of the downregulated hub genes in HDAC11-overexpressing CMSCs are closely related to translational and post-translational processes. For example, RPL7 is a component of the large ribosome subunit essential for protein synthesis [77], EEF1B2 is a guanine nucleotide exchange factor that facilitates the transfer of aminoacylated tRNAs to ribosomes [78], and PCMTD1 is involved in protein ubiquitination and degradation [51]. These findings suggest that HDAC11 is highly involved in the regulation of protein synthesis and degradation pathways. Additionally, the downregulation of CLK1, a kinase involved in pre-mRNA processing [79], indicates that HDAC11 may affect RNA alternative splicing in CMSCs, consistent with previous reports linking HDAC11 to the survival of motor neurons via mRNA splicing regulation [80].

Long non-coding RNAs (lncRNAs) play a critical role in regulating key functions in heart development and regeneration, such as myofibril maturation, electrophysiology, calcium handling, metabolic development, cardiomyocyte proliferation, and cardiac regeneration [81,82]. However, the functions of HDAC11-responsive lncRNAs in CMSCs remain largely unexplored. Our RNA-seq analysis revealed GM11944, GM11266, GM4123, and GM32569 were downregulated in Ad-HDAC11-treated CMSCs. The downregulation of GM11266 in Ad-HDAC11-treated CMSCs was further confirmed by qRT-PCR. GM11266 is located on chr4:82,153,892-82,193,196 and contains two loci bound by histone 3.3 [83]. The functional implications of the HDAC11-mediated regulation of GM11266 and its potential impact on CMSC function remain unknown, providing an intriguing direction for future study.

## 5. Conclusions

HDAC11 overexpression in CMSCs induces significant transcriptional reprogramming, characterized by the upregulation of genes involved in cell cycle regulation and proliferation, and the downregulation of genes linked to translational and post-translational mechanisms. HDAC11 overexpression also activated pathways related to chromosome organization and other critical processes for cell cycle progression and proliferation, while suppressing pathways involved in nucleotide transport and phagolysosome formation.

## 6. Future Perspective

Cardiac mesenchymal stem cells (CMSCs) can support endogenous cardiac repair through paracrine mechanisms, such as the secretion of pro-angiogenic cytokines and extracellular vesicles, including exosomes [84,85,86]. Following myocardial infarction, cardiac stem cells are rapidly depleted in both infarcted and non-infarcted regions, as reported by Mouquet et al. [28]. While stem cell transplantation holds promise for replenishing lost cardiac stem cells and restoring function, major challenges remain—particularly early post-transplant cell loss and low cell retention/survival [87]. Our findings highlight the potential of enhancing heart repair through the novel strategy of overexpressing HDAC11 in endogenous CMSCs to promote their proliferation and regenerative activity.

## Data Availability

The RNA-seq data are available upon request.

## Supplementary Materials

The following supporting information can be downloaded at: https://www.mdpi.com/article/10.3390/biom15050662/s1, All Western blot original images.
