# Supplementary figures and images for: Identification of Critical Molecular Pathways Induced by HDAC11 Overexpression in Cardiac Mesenchymal Stem Cells
Source: Biomolecules. 2025 May 3;15(5):662. doi: 10.3390/biom15050662 (PMC12109384; doi:10.3390/biom15050662)

Figure 1:

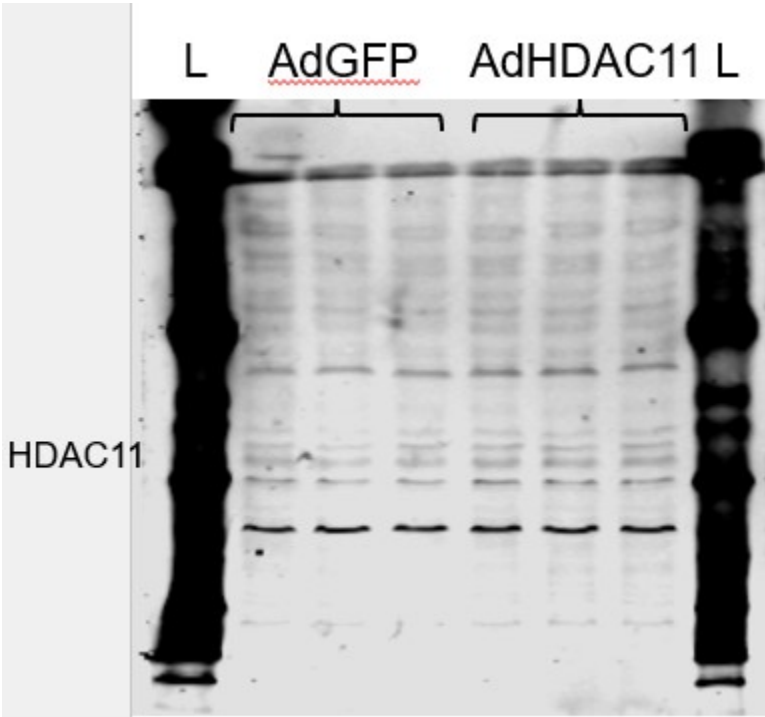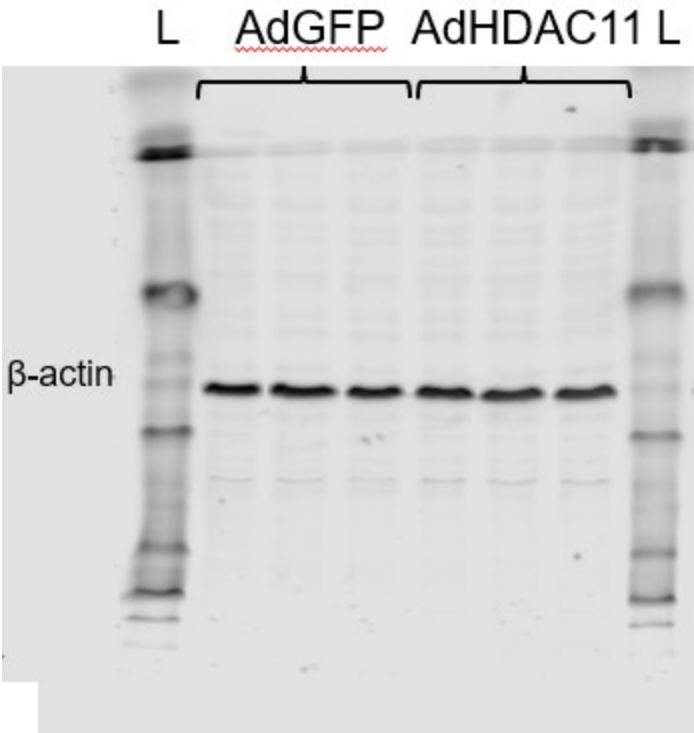

Figure 6:

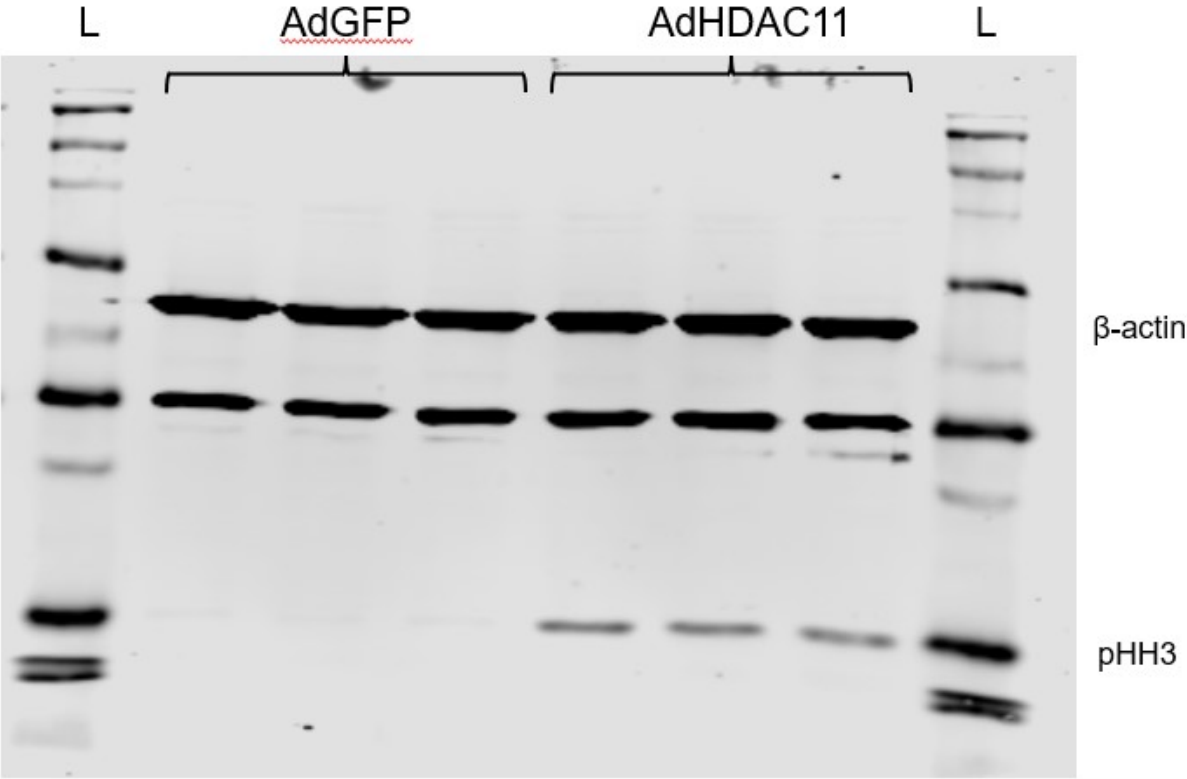

Supplement: Supplementary file 1 [file biomolecules-15-00662-s001.zip › biomolecules-3553681-supplementary.pdf]
